# Posterior Malleolar Fracture Assessment: An Independent Interobserver and Intraobserver Validation of Three Computed Tomography-Based Classifications

**DOI:** 10.5435/JAAOSGlobal-D-22-00258

**Published:** 2023-01-09

**Authors:** Sergio Morales, Jafet Massri-Pugin, Pablo Mery, Joaquín Palma, Jorge Filippi, Andrés Villa

**Affiliations:** From the Department of Orthopaedic Surgery, School of Medicine, Pontificia Universidad Católica de Chile, Santiago, Chile.

## Abstract

Posterior malleolus fractures occur in up to 50% of all ankle fractures. Several classification systems exist for their characterization, especially under CT. However, those classifications do not report the level of agreement or do it incompletely. This study aims to independently assess three posterior malleolus fracture classifications (Haraguchi, Bartoníček/Rammelt, and Mason).

**Methods:**

This study was designed according to the Guidelines for Reporting Reliability and Agreement Studies. Ninety-four CT scans of patients with ankle fractures that had posterior malleolus fractures were included. Posterior malleolus fractures were assessed by six evaluators (three attending foot and ankle surgeons and three orthopaedic surgery residents) according to Haraguchi, Bartoníček/Rammelt, and Mason classifications. All images were reassessed by the same evaluators in a random sequence 3 weeks later. The kappa (*k*) coefficient was used to determine the interobserver and intraobserver agreement. Statistical significance was established using *P* < 0.05 with a 95% confidence interval (CI).

**Results:**

The interobserver agreement was moderate for Haraguchi, Bartoníček/Rammelt, and Mason classifications with a global *k* value of 0.52 (95% CI, 0.43 to 0.60), 0.53 (95% CI, 0.46 to 0.61), and 0.54 (95% CI, 0.47 to 0.62), respectively. The intraobserver agreement was substantial for Haraguchi, Bartoníček/Rammelt, and Mason classifications, with an overall *k* value of 0.70 (95% CI, 0.64 to 0.74), 0.73 (95% CI, 0.68 to 0.78), and 0.73 (95% CI, 0.69 to 0.78), respectively. Interobserver and intraobserver agreement among orthopaedic surgeons and residents had no significant difference.

**Conclusion:**

The current classifications for posterior malleolus fractures showed a substantial intraobserver agreement. Nevertheless, the interobserver agreement obtained was just moderate for all classifications, independent of the level of expertise of the evaluators.

Posterior malleolus fractures are located in the posterior articular border of the distal tibia. They can occur in isolation but are most commonly associated with medial and lateral malleolus fractures. Up to 50% of all ankle fractures have a fracture of the posterior malleolus,^1^ and its presence could determine poor functional outcomes in the short and medium terms.^2,3^

Anterior-posterior, lateral, and mortise ankle radiograph views are the primary approach for diagnosing ankle fractures. However, several studies have demonstrated that radiographs are less accurate than CT scans in the setting of posterior malleolus fracture.^4,5^ Thus, a CT scan is preferred as a diagnostic tool for posterior malleolus fractures.

The first classification using a CT scan was conducted by Haraguchi et al.^6^ (Figure 1). Using only axial images, they proposed three types of posterior malleolus fractures:

-Type I: Posterolateral oblique
-Type II: Medial extension
-Type III: Small shell

**Figure 1:**
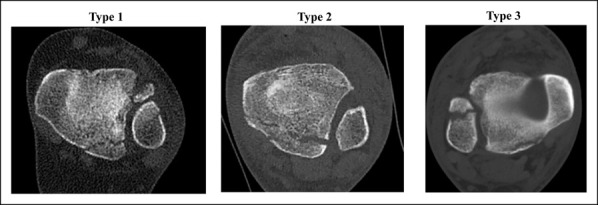
Images demonstrating the types of Haraguchi classification.

In 2015, Bartoníček and Rammelt et al.^7^ (Figure 2) proposed a new classification using axial, coronal, sagittal, and 3D reconstruction images. They categorized the fracture of the posterior malleolus into five types:

-Type I: Extraincisural fragment with an intact incisura
-Type II: Posterolateral fragment involving the incisura
-Type III: Posteromedial two-part fragment extended to the medial malleolus
-Type IV: Large posterolateral triangular fragment
-Type V: An irregular osteoporotic fracture not classifiable in the other types

**Figure 2:**
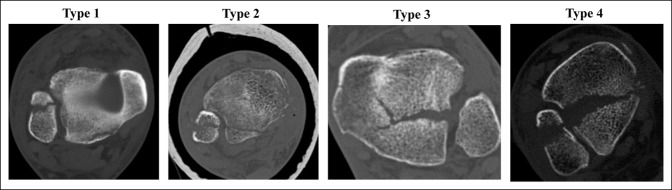
Images demonstrating the types of Bartoníček and Rammelt classification.

None of the two abovementioned classifications reported the interobserver and intraobserver agreement.^6,7^ Furthermore, they do not describe a treatment algorithm based on the fracture type.

Finally, Mason et al.^8^ (Figure 3) characterized the posterior malleolus into four categories:

-Type I: A small extra-articular posterior malleolar fragment
-Type IIA: A posterolateral triangular fragment involving the incisura fibularis
-Type IIB: A posterolateral and posteromedial fragment at a 45° angle to the primary fragment
-Type III: A coronal plane fracture involving the entire posterior plafond

**Figure 3:**
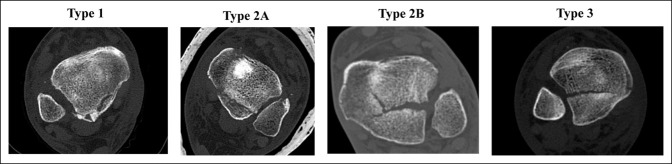
Images demonstrating the types of Mason classification.

The authors also proposed a specific surgical approach depending on the classification. Although they reported an interobserver kappa (*k*) Cohen value of 0.919 (between two fellowship-trained foot and ankle surgeons), they did not report the intraobserver agreement.^8^

Kottner et al.^9^ published 15 items in the Guidelines for Reporting Reliability and Agreement Studies (GRRAS) in an attempt to improve the quality of reliability and agreement studies. Current classifications of posterior malleolus fractures do not report (or report incompletely) the level of agreement and reliability, limiting their widespread use. In addition, no studies have compared the CT-based classifications for posterior malleolus fracture according to the mentioned guidelines.^9^ Furthermore, it is uncertain whether agreement differs between physicians with different levels of expertise for these classifications. Therefore, this study aimed to conduct independent research to compare the level of agreement of Haraguchi, Bartoníček/Rammelt, and Mason classifications^6-8^ among physicians with different levels of orthopaedic training in the management of posterior malleolus fracture.

## Methods

We used the GRRAS to conduct this study.^9^ Approval from the institutional review board was obtained. One of the authors, who did not participate in the assessment of the cases, did a retrospective search of patients with ankle fractures surgically treated between 2013 and 2020 in our institution. Inclusion criteria were patients 18 years and older who had a preoperative ankle CT scan with a posterior malleolus fracture. CT scans had to have axial and sagittal image views available. Exclusion criteria were history of ankle fracture, surgery, tumors, or infections. In addition, posterior pilon fractures, defined by Bartonicek et al., were excluded.^7^ Ninety-four cases were finally chosen for the analysis.

CT images were evaluated using the Impax Web3000 program (Agfa-Gevaert) by six independent evaluators: three attending orthopaedic foot and ankle surgeons and three last year orthopaedic surgery residents. Evaluators were trained in the posterior malleolar classifications described by Haraguchi, Bartoníček/Rammelt, and Mason through an online education session. Original articles of each classification were available at the evaluation stage to be used in the case of doubts during the assessment.

Each evaluator classified the fracture of the posterior malleolus according to the Haraguchi classification into type I, II, or III; Bartoníček/Rammelt classification into type I, II, III, IV, or V; and Mason classification into type I, IIA, IIB or III.^6‐8^ CT images were reassessed in a random sequence 3 weeks later by the same evaluators to avoid recall bias. The new arrangement of CT images was done using the randomization tool of Microsoft Excel by an author who did not participate in classifying the cases. Interobserver agreement was obtained by comparing the first response of each evaluator. Intraobserver agreement was calculated by comparing each evaluator's first and second evaluations for the same case. Both interobserver and intraobserver agreements were calculated for each classification. The sample was selected by convenience (convenience sampling technique), including all trimalleolar fractures receiving surgical treatment between 2013 and 2022 in a university hospital. The sample size was not calculated because of the absence of the reported *k* coefficient in previous studies of the posterior malleolus.

Stata software 14.2 (StataCorp) was used for statistical analysis. *T*-tests were conducted to detect any significant difference in the prevalence of each classification between the original publications and our study. Cohen and Fleiss *k* coefficients with their respective 95% confidence intervals (CIs) were used for determining the interobserver and intraobserver agreement, respectively. Levels of agreement for *k* were estimated as proposed by Landis and Koch.^10^ *K* values of 0.00 to 0.20 were considered slight agreement; 0.21 to 0.40, fair agreement; 0.41 to 0.60, moderate agreement; 0.61 to 0.80, substantial agreement; and 0.81 to 1.00, almost perfect agreement.

## Results

Six observers evaluated 94 cases and did 564 assessments for each classification. They repeated the process 3 weeks after the first evaluation. Sixty-five cases (69.14%) of the sample had a 3D reconstruction available for analysis.

We did not detect any significant difference in the prevalence for each injury type between the original classification studies and our study. Table 1 presents the respective percentages of each fracture type for the three classifications, means, and the corresponding p values.

**Table 1:** Distribution of Responses for Each Type of the Classification of Haraguchi, Bartoníček/Rammelt, and Mason

Classifications and Subtypes	Original study prevalence, N (%)	Current study prevalence, N (%)	*P* value
Haraguchi			
Type I	38 (67.0)	304 (53.9)	0.12
Type II	11 (19.0)	167 (29.6)	0.68
Type III	8 (14.0)	93 (16.5)	0.85
Total	57	564	
Bartoníček/Rammelt			
Type I	11 (8.0)	138 (24.5)	0.21
Type II	74 (52.0)	227 (40.2)	0.07
Type III	39 (28.0)	152 (27.0)	0.90
Type IV	13 (9)	42 (7.4)	0.85
Type V	4 (3.0)	5 (0.9)	0.81
Total	141	564	
Mason			
Type I	41 (34.0)	144 (25.5)	0.28
Type IIA	30 (24.0)	52 (9.2)	0.06
Type IIB	25 (21.0)	223 (39.5)	0.07
Type III	25 (21.0)	145 (25.7)	0.61
Total	121	564	

Values for original and current studies are presented.

### Interobserver Agreement

The overall interobserver agreement was moderate for all classifications (see Table 2), with a *k* value of 0.52 (95% CI, 0.43 to 0.61) for Haraguchi, 0.53 (95% CI, 0.46 to 0.61) for Bartoníček/Rammelt, and 0.54 (95% CI, 0.47 to 0.62) for Mason classifications, without significant differences between them. The percentage of agreement was 71% for the Haraguchi classification (95% CI, 66% to 77%), 67% for the Bartoníček/Rammelt classification (95% CI, 62% to 72%), and 68% for the Mason classification (95% CI, 63% to 73%). For all types in the Haraguchi classification, the interobserver agreement was moderate. Using the Bartoníček/Rammelt classification, the interobserver agreement was moderate for types I, II, and III; substantial for type IV; and no agreement for type V. The classification of Mason showed moderate agreement for types I, IIB, and III and substantial agreement for type IIA.

**Table 2:** Interobserver Agreement (*k*) According to the Level of Expertise

	Fleiss Kappa (95% CI)		
Classification	Orthopaedic Residents	Foot and Ankle Surgeons	Overall
Haraguchi	0.56 (0.44-0.68)	0.54 (0.43-0.65)	0.52 (0.43-0.61)
Bartoníček/Rammelt	0.54 (0.43-0.64)	0.53 (0.42-0.63)	0.53 (0.46-0.61)
Mason	0.53 (0.43-0.64)	0.54 (0.44-0.64)	0.54 (0.47-0.62)

There was no difference in the interobserver agreement between attending surgeons and residents for all classifications. The overall *k* coefficients for each classification type according to the level of expertise are presented in Table 2, and *k* coefficients for each fracture within each classification are presented in Table 3.

**Table 3:** Interobserver Agreement (*k*) for Each Fracture Type

Classification/Type	Fleiss Kappa (*k*)	95% CI
Haraguchi		
I	0.53	0.48-0.58
II	0.55	0.50-0.60
III	0.45	0.40-0.50
Bartoníček/Rammelt		
I	0.45	0.40-0.50
II	0.53	0.48-0.58
III	0.58	0.53-0.63
IV	0.73	0.68-0.78
V	−0.01	−0.07 to 0.05
Mason		
I	0.47	0.42-0.52
IIA	0.68	0.63-0.73
IIB	0.54	0.49-0.59
III	0.55	0.50-0.60

### Intraobserver Agreement

The overall intraobserver agreement was substantial for all classifications (Table 4), with a *k* value of 0.70 (95% CI, 0.64 to 0.75) for the Haraguchi classification, 0.73 (95% CI, 0.69 to 0.78) for the Bartoníček/Rammelt classification, and 0.73 (95% CI, 0.69 to 0.78) for the Mason classification, without significant differences between them; full agreement was obtained in 82% of cases of the Haraguchi classification (95% CI, 79% to 85%), in 82% for the Bartoníček/Rammelt classification (95% CI, 78% to 85%), and in 81% for the Mason classification (95% CI, 78% to 85%). No difference was found in the intraobserver agreement between attending surgeons and residents. The detailed *K* coefficients and respective 95% CIs according to the level of expertise are presented in Table 4.

**Table 4:** Intraobserver Agreement (*k*) According to the Level of Expertise

	Cohen Kappa (95% CI)		
Classification	Orthopaedic Residents	Foot and Ankle Surgeons	Overall
Haraguchi	0.66 (0.59-0.74)	0.73 (0.66-0.80)	0.70 (0.64-0.75)
Bartoníček/Rammelt	0.66 (0.59-0.73)	0.80 (0.74-0.86)	0.73 (0.69-0.78)
Mason	0.67(0.60-0.75)	0.78 (0.72-0.84)	0.73 (0.69-0.78)

## Discussion

This study showed moderate interobserver and substantial intraobserver agreement for Haraguchi, Bartoníček/Rammelt, and Mason classifications. None of these classifications showed superiority over the others in the interobserver and intraobserver agreement. To the best of our knowledge, this is the first study that independently assessed the most used classifications for posterior malleolus ankle fractures under a CT scan using the GRRAS, proposed by Kottner et al.^9^

Kleinertz et al.,^11^ recently published on this subject with the same classifications. They used four observers and showed substantial, if not perfect, interobserver and intraobserver reliabilities independent of the observer's level of expertise. They did not report on the prevalence of each type of fracture, so we cannot compare our cohort for the complexity of the fractures that were analyzed. They stated in the study that the group thoroughly discussed the method, so maybe their observers were more familiar with the classifications than ours, who only had one online capacitation session.

Previous reliability and reproducibility studies have suggested that the observers' experience improves the reliability of a classification system.^12-14^ However, we found no difference in the interobserver agreement, regardless of the expertise of the observers for all classifications. This phenomenon has already been reported in recent foot and ankle reliability studies.^15^ Certain attributes of any classification system are desirable, such as being reproducible and replicable,^16^ not only by the authors who developed them. We performed an independent analysis in a different setting; for this reason, our results contribute to determining the external validity of the analyzed classifications.

Preoperative evaluation of trimalleolar fractures must include a CT scan, considering that plain radiographs cannot interpret the morphology of posterior malleolus.^17^ Moreover, CT scans could modify surgical planning and patient positioning in the operating room.^18,19^ No information about CT 3D reconstruction has been reported on this topic; moreover, it is unclear whether there is some benefit in improving the level of agreement among the evaluators.

Haraguchi et al.^6^ published the pioneer publication of posterior malleolus ankle fractures under a CT scan. They evaluated 57 patients and demonstrated that the most prevalent fracture was the oblique posterolateral (type I) in 67% of the cases, followed by the fracture type with medial extension in 19% of cases. Only 14% of cases exhibited a small shell–type fracture. However, they did not report how many raters were involved, their training, or how the sample size was calculated. Importantly, they did not report any estimate of reliability and agreement. Although the study by Haraguchi et al.^6^ allowed a better understanding of the anatomy of the posterior malleolus, its shortcomings are evident. Our study showed similar results in the prevalence of each injury pattern but a moderate interobserver agreement for this classification, preventing its massive use.

In 2015, Bartoníček and Rammelt et al.^7^ created their classification for posterior malleolus ankle fractures under a CT scan, including 3D reconstruction in most patients if available. Two senior authors independently evaluated the CT scan of 137 patients with Weber B or C ankle fractures with a mean age of 49 years (range 19 to 83). If no consensus was obtained, both evaluators reviewed the CT scans together. In that study, the most common injury was Type II (posterolateral fragment with extension into the incisura fibularis). Type II was observed in 50.0% of cases, followed by a posteromedial two-part fragment (Type III) in 28% of cases. Type V was the least frequent, with a prevalence of 3%. In that study,^7^ the authors did not perform an agreement and reliability analysis. In general, this is our institution's most used classification for posterior malleolus ankle fractures. When comparing the original publication of Bartoníček/Rammelt^7^ and our study, we did not detect significant differences in the prevalence of each injury type. However, we obtained an overall k value of 0.53 for interobserver agreement,^20^ which is lower than the threshold suggested for using a classification.

In 2017, Mason et al.^8^ described a classification based on CT scans that tried to explain the pathomechanics of posterior malleolus. Two blinded foot and ankle surgeons evaluated 121 patients with a mean age of 48 years (range 17 to 90). Compared with Haraguchi and Bartoníček/Rammelt classifications,^6,7^ this is a unique classification system that progresses in severity. Nevertheless, no clinical studies have demonstrated that type III has worse outcomes than type II or I. Therefore, we consider that, until proven otherwise, the classification of Mason is not quantitative. Another important aspect is that the Mason classification was conducted with an agreement analysis, reporting almost perfect interobserver agreement (*k* 0.92), a higher value than the one obtained in our study (*k* 0.54). The main reason to explain the difference is that there is evidence that independent assessments of classifications exhibit lower agreement than original studies.^12,15^ Given that no replicate measurements were performed in the study of Mason et al.,^8^ the intraobserver agreement was not calculated. Although we consider that the publication of Mason gives more information about the methodology and could suggest the management of these fractures, the level of agreement obtained in our study does not guarantee a reliable use in the clinical and research fields. It is necessary to mention that our raters do not use this classification in clinical practice because there is evidence that being familiar with some classification systems could improve reliability.^16^ In addition, it could be hypothesized that our results may be influenced by different patterns of fractures or different prevalence of each fracture type. We compared proportions for each subtype fracture for all classifications in our sample matched with proportions described in original studies, finding no statistical significance, so the samples are comparable.

When all three classifications are compared, it is clear that some similarities exist based on descriptions. It is necessary to remember that all those classifications were arbitrarily chosen by morphology assessment. Because of the lack of physiopathological studies, these types might not be different. For example, a prior study on posterior malleolus fracture suggests that type III and type I of Haraguchi classification may be a continuous spectrum.^21^ We believe that an ideal classification of posterior malleolus fracture should include a physiopathological or severity pathway to contribute to clinical decision making.

This study has some weaknesses to take into account. First, the sample size was selected for convenience. One of the disadvantages of this process is that it can over or underrepresent certain groups of patients within the sample size. However, this nonprobability method allowed us to select patients with a trimalleolar ankle fracture, often surgically treated because they are considered unstable. Second, this independent study was not multicentric, which is recommended during a validation process of any classification.^22^ Third, we do not have precise information for explaining why only some patients had a 3D reconstruction, confirming a selection bias that could not be addressed. The strength of this study is that it was conducted following the GRRAS.^9^ Moreover, the level of agreement was obtained using six evaluators with different levels of expertise. This independent study provided insights into the shortcomings of the current classifications of posterior malleolus ankle fractures. Future studies are necessary to formulate a new classification under strict guidelines, which gives orientation to the surgeon during the decision-making process.

## Conclusion

This is the first agreement and reliability study that compares the three most used classifications for posterior malleolus ankle fractures, according to the GRRAS. Our results showed substantial intraobserver agreement but only moderate interobserver agreement for all classifications, limiting adequate understanding and communication in the clinical and research fields. These findings did not change, regardless of the level of experience of the evaluators. Owing to a moderate level of agreement and considering that no classification is superior to the others, we estimate the need to generate a new classification for posterior malleolus ankle fractures that gives a higher level of agreement, treatment, and prognosis orientation of these fractures.

## References

[1] Switaj PJ Weatherford B Fuchs D Rosenthal B Pang E Kadakia AR: Evaluation of posterior malleolar fractures and the posterior pilon variant in operatively treated ankle fractures. Foot Ankle Int 2014;35:886-895. 24942618 10.1177/1071100714537630

[2] Tejwani NC Pahk B Egol KA: Effect of posterior malleolus fracture on outcome after unstable ankle fracture. J Trauma Inj Infect Crit Care 2010;69:666-669. 10.1097/TA.0b013e3181e4f81e 20838137

[3] Abarquero-Diezhandino A Luengo-Alonso G Alonso-Tejero D Sánchez-Morata EJ Olaya-Gonzalez C Vilá y Rico J: Study of the relation between the posterior malleolus fracture and the development of osteoarthritis. Rev Esp Cir Ortop Traumatol (Engl Ed) 2020;64:41-49. 31676414 10.1016/j.recot.2019.09.002

[4] Büchler L Tannast M Bonel HM Weber M: Reliability of radiologic assessment of the fracture anatomy at the posterior tibial plafond in malleolar fractures. J Orthop Trauma 2009;23:208-212. 19516096 10.1097/BOT.0b013e31819b0b23

[5] Ferries JS DeCoster TA Firoozbakhsh KK Garcia JF Miller RA: Plain radiographic interpretation in trimalleolar ankle fractures poorly assesses posterior fragment size. J Orthop Trauma 1994;8:328-331. 7965295 10.1097/00005131-199408000-00009

[6] Haraguchi N Haruyama H Toga H Kato F: Pathoanatomy of posterior malleolar fractures of the ankle. J Bone Joint Surg 2006;88:1085-1092. 16651584 10.2106/JBJS.E.00856

[7] Bartoníček J Rammelt S Kostlivý K Vaněček V Klika D Trešl I: Anatomy and classification of the posterior tibial fragment in ankle fractures. Arch Orthop Trauma Surg 2015;135:505-516. 25708027 10.1007/s00402-015-2171-4

[8] Mason LW Marlow WJ Widnall J Molloy AP: Pathoanatomy and associated injuries of posterior malleolus fracture of the ankle. Foot Ankle Int 2017;38:1229-1235. 28758439 10.1177/1071100717719533

[9] Kottner J Audige L Brorson S : Guidelines for reporting reliability and agreement studies (GRRAS) were proposed. Int J Nurs Stud 2011;48:661-671. 21514934 10.1016/j.ijnurstu.2011.01.016

[10] Landis JR Koch GG: The measurement of observer agreement for categorical data. Biometrics 1977;33:159-174. 843571

[11] Kleinertz H Mueller E Tessarzyk M Frosch KH Schlickewei C: Computed tomography-based classifications of posterior malleolar fractures and their inter- and intraobserver reliability: A comparison of the Haraguchi, bartoníček/rammelt, and Mason classifications. Arch Orthop Trauma Surg 2022;142:3895-3902. 35094134 10.1007/s00402-021-04315-y

[12] Urrutia J Zamora T Yurac R : An independent interobserver reliability and intraobserver reproducibility evaluation of the new AOSpine Thoracolumbar Spine Injury Classification System. Spine 19762015;40:E54-E58. 10.1097/BRS.0000000000000656 25341990

[13] Urrutia J Zamora T Yurac R : An independent inter- and intraobserver agreement evaluation of the AOSpine subaxial cervical spine injury classification system. Spine (Phila Pa 1976) 2017;42:298-303. 26630415 10.1097/BRS.0000000000001302

[14] Schipper IB Steyerberg EW Castelein RM Vugt ABV: Reliability of the AO/ASIF classification for pertrochanteric femoral fractures. Acta Orthop Scand 2001;72:36-41. 11327411 10.1080/000164701753606662

[15] Palma J Villa A Mery P : A new classification system for pilon fractures based on CT scan: An independent interobserver and intraobserver agreement evaluation. J Am Acad Orthop Surgeons 2020;28:208-213. 10.5435/JAAOS-D-19-00390 31800439

[16] Ramappa M Bajwa A Singh A Mackenney P Hui A Port A: Interobserver and intraobserver variations in tibial pilon fracture classification systems. Foot 2010;20:61-63. 20609577 10.1016/j.foot.2010.06.002

[17] Meijer DT Doornberg JN Sierevelt IN : Guesstimation of posterior malleolar fractures on lateral plain radiographs. Injury 2015;46:2024-2029. 26253385 10.1016/j.injury.2015.07.019

[18] Kumar A Mishra P Tandon A Arora R Chadha M: Effect of CT on management plan in malleolar ankle fractures. Foot Ankle Int 2018;39:59-66. 29232164 10.1177/1071100717732746

[19] Donohoe S Alluri RK Hill JR Fleming M Tan E Marecek G: Impact of computed tomography on operative planning for ankle fractures involving the posterior malleolus. Foot Ankle Int 2017;38:1337-1342. 28954524 10.1177/1071100717731568

[20] Sanders RW: The problem with apples and oranges. J Orthop Trauma 1997;11:465-466.

[21] Mangnus L Meijer DT Stufkens SA : Posterior malleolar fracture patterns. J Orthop Trauma 2015;29:428-435. 25785358 10.1097/BOT.0000000000000330

[22] Audigé L Bhandari M Hanson B Kellam J: A concept for the validation of fracture classifications. J Orthop Trauma 2005;19:401-406. 16003200 10.1097/01.bot.0000155310.04886.37

